# Exploration of the postponing mechanism that delays carcinoma onset

**DOI:** 10.1186/1475-2867-10-38

**Published:** 2010-10-22

**Authors:** Chao-Nan Qian

**Affiliations:** 1 Department of Nasopharyngeal Carcinoma, Sun Yat-Sen University Cancer Center, 651 Dongfeng East Road, Guangzhou 510060, PR China; 2 State Key Laboratory of Oncology in South China, Sun Yat-Sen University Cancer Center, 651 Dongfeng East Road, Guangzhou 510060, PR China

## Abstract

The average age at onset of malignancies arising from epithelial tissues is between 40 and 70 years old even in familial cancers. Although it is believed that the accumulation of multiple genetic alterations is needed for cancer onset, we hypothesize--based on the diversity of ages at onset for most types of epithelial cancer--that there is a postponing mechanism inside the human body that significantly delays the process of carcinogenesis. The key molecules controlling the cancer onset, here called "postponers", are hypothesized to be functioning in the individuals carrying susceptibility genes. As a consequence, cancers occur in middle age or even old age, with several decades of cancer-free lifetime for the patient. Genome-wide association studies and genomic expression profiling are suggested to identify candidate postponers. Hypothetic gene expression patterns for identifying candidate postponers are illustrated. Animal models will be helpful to test whether the absence or presence of the postponer molecules can alter the onset age of spontaneous tumors. If this hypothesis is true, by amplification of the postponing mechanism we might be able to significantly delay the onset of tumors, so that individuals carrying cancer susceptibility traits could gain an additional significant period of cancer-free life. Moreover, destructive prophylactic surgeries, e.g., for women who have *BRCA1/2 *gene mutations, might be avoided.

## Introduction

Hereditary susceptibility has been increasingly recognized to be an important etiological factor in many cancers. Familial aggregation is one of the phenotypes reflecting the contribution of genetic factors in carcinogenesis [1-4]. Genetic influences on cancer onset can also present as geographic and racial aggregation in some cancers. For example, nasopharyngeal carcinoma (NPC) has the highest incidence rate in southern China and Southeast Asia in populations with similar racial/ethnic backgrounds [5,6].

In a study comparing the age of cancer onset, Brandt and colleagues found that cancers in individuals with a family history occur 2.6-16.3 years earlier than in sporadic patients [7]. The early onset in familial cases is observed in a variety of malignancies, including leukemia, non-Hodgkin lymphoma, and cancers arising in the cervix, breast, nasopharynx, ovary, nervous system, colorectum, endometrium, bladder, lung, prostate, skin, stomach, and pancreas [7,8]. These findings strengthen the notion that genetic susceptibility plays an important role in the carcinogenesis of a variety of cancers.

Thanks to advances in molecular technology, especially genome-wide association studies (GWAS), one could expect that in the near future susceptibility gene(s) could be identified in more malignancies [9]. However, another important question is then raised: if the genetic trait is the main culprit, why does it show up so late for the most common cancers arising in epithelial tissue, with the average age of onset being 40-70 years old even in the familial cancers, although the affected individual carries the gene(s) from day 1 of his/her life?

The commonly accepted explanation for the delayed onset of cancer is that accumulation of the genetic alterations is needed for carcinogenesis. The multiple mutation theory has been widely acknowledged for tumor initiation [10]. Genetic variety inside a solid tumor correlating with different invasiveness of the cells [11] further supports the theory that multiple genetic alterations might be needed for cancer formation and progression. If the accumulation of multiple genetic alterations is needed, the remarkable variety of age of onset within each type of epithelial cancer suggests that the speed of this genetic accumulation is different, probably controlled by a molecular postponing system functioning at different efficiencies. On another hand, the multiple mutation theory is also challenged by the fact that a particular type of cancer only develops in a small proportion of a population heavily exposed to known chemical carcinogens [12], suggesting that a postponing mechanism could be functioning to prevent tumorigenesis in the lifetime of the exposed population.

Another hypothetical explanation for the delayed onset of cancer is that the immune system in the patient prevents the disease onset in his/her childhood and adolescence. However, most cancer patients, especially those with early-stage disease, do not show impaired immune function, weakening the rationale of the explanation. Other unknown mechanism(s) in human body may be acting to delay the cancer onset.

## Presentation of hypothesis

Herein, I hypothesize that there is a postponing mechanism that significantly delays the process of epithelial carcinogenesis. The key molecules controlling cancer onset, "postponers", are hypothesized to be functioning in individuals carrying susceptibility genes. As a consequence, cancers occur in middle age or even old age, with several decades of cancer-free lifetime for the patients.

## Testing the hypothesis

To test this hypothesis, comprehensive studies are needed. The first task is to identify candidate postponer molecules. Population-based GWAS might be helpful in identifying candidates by comparison of the genetic differences between cancer patients and healthy controls, and among the cancer patients with different ages of onset.

**Figure 1:**
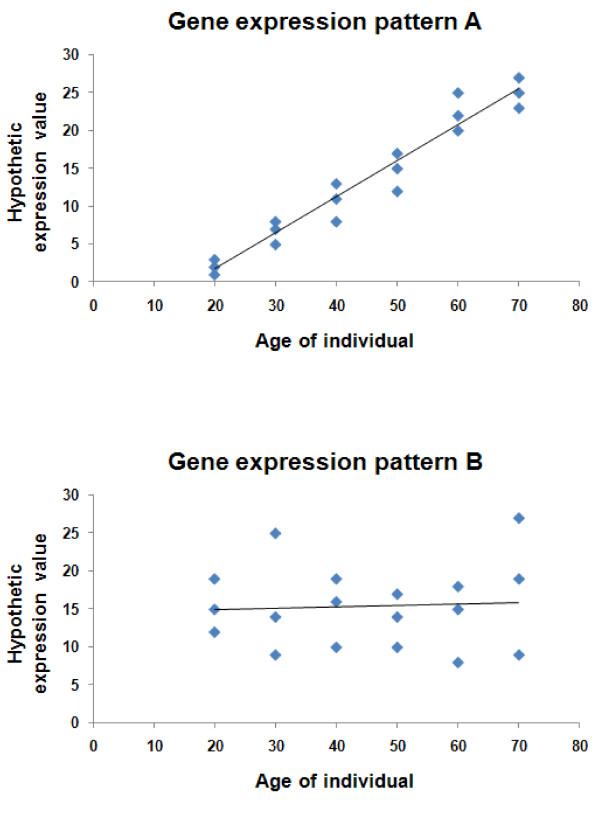
**Hypothetic gene expression patterns in cancer patients and healthy controls in a population with the similar genetic background**. If both groups of cancer patients and healthy controls have a gene expressed in Pattern A, we can assume that this gene might correlate to normal aging but not cancer onset. However, if a gene expression only correlates with the age of disease onset in cancer patients (Pattern A) but does not correlate with normal aging in healthy controls (Pattern B), we can assume that this gene might correlate to the postponing of cancer onset.

Another approach to identifying postponer candidates might be quantitative comparison of genomic expression profiling among the tumors manifesting in patients of different ages. For this strategy, the experiments should be wisely designed to exclude the confounding factors generated from normal aging. Figure 1 demonstrates hypothetic patterns of gene expression that can provide us some clues for identifying postponer candidate.

In vitro functional studies to confirm the biological impacts of the identified postponer candidates would be critical. Numerous approaches to evaluate the roles of the candidate molecules on carcinogenesis, apoptosis, senescence, and autophagy should be considered.

Appropriate animal models will be useful for testing whether the presence or absence of the postponer candidates can alter the onset age of spontaneous tumors. In an animal model with homozygous *Pten *deletion in the renal epithelia using the Cre-lox system, typical renal pelvic transitional cell carcinoma (urothelial carcinoma) can be induced in old-age animals [13]. Although the animals harbor the genetic alteration from their inception, the tumor incidence is 18.2% in mice younger than 6 months and increases to 57.1% in mice older than 1 year. The late tumor occurrence of this animal model is consistent with human renal pelvic transitional cell carcinoma, in which the average age of tumor onset is 70 years old [14]. Therefore, this is one of the appropriate models to test whether knock-out of postponer candidate(s) can expedite cancer onset.

## Implications of hypothesis

The identification of postponer molecules is important, especially for personalized medicine aiming to provide high quality of life to cancer patients. For example, aggressive and destructive prophylactic surgical approaches, including bilateral mastectomy and oophorectomy, have been recommended for women with the *BRCA1/2 *gene mutation [15,16]. If the effect of a postponing mechanism for breast cancer can be amplified in these high-risk individuals to gain a significant period of cancer-free time (or even their entire lifespan), these surgeries might then be avoided.

Proving this hypothesis will also be helpful for improving cancer patients' quality of life. In NPC, for example, as hereditary factor(s) are identified in the future, the carriers of genetic susceptibility might bear huge psychological burdens due to lack of effective preventive approaches within current medical practice. Strengthening or amplifying the postponing mechanism might be the hope of these individuals.

## List of abbreviations

GWAS: genome-wide association studies; NPC: nasopharyngeal carcinoma
